# MEP-YOLOv5s: Small-Target Detection Model for Unmanned Aerial Vehicle-Captured Images

**DOI:** 10.3390/s25113468

**Published:** 2025-05-30

**Authors:** Shengbang Zhou, Song Zhang, Chuanqi Li, Shutian Liu, Dong Chen

**Affiliations:** 1 Guangxi Key Laboratory of Functional Information Materials and Intelligent Information Processing, Nanning Normal University, Nanning 530001, China; 2 Guangxi Geographical Indication Crops Research Center of Big Data Mining and Experimental Engineering Technology, Nanning Normal University, Nanning 530001, China; 3 Guangxi Key Laboratory of Earth Surface Processes and Intelligent Simulation, Nanning Normal University, Nanning 530001, China

**Keywords:** small object detection, UAV, feature extraction, multi-scale attention

## Abstract

Due to complex backgrounds, significant scale variations of targets, and dense distributions of small objects in Unmanned Aerial Vehicle (UAV) aerial images, traditional object detection algorithms face challenges in adapting to such scenarios. This article introduces a drone detection model, MEP-YOLOv5s, which optimizes the Backbone, Neck layer, and C3 module based on YOLOv5s, and combines effective attention mechanisms to improve the training efficiency of the model by replacing the traditional CIoU loss (Complete Intersection over Union) with MPDIoU (Minimum Point Distance-based Intersection over Union) loss. This model demonstrates an excellent performance in handling typical drone detection scenarios, especially for small and dense objects. To holistically balance the detection accuracy and inference efficiency, we propose a Comprehensive Performance Indicator (CPI), which evaluates the model performance by considering both accuracy and efficiency. Evaluations on the VisDrone2019 dataset demonstrate that MEP-YOLOv5s achieves a 3.3% improvement in precision (P), a 20.9% increase in mAP@0.5, and a 19.86% gain in the CPI (α = 0.5) compared with the baseline model. Additional experiments on the NWPU VHR-10 dataset confirm that MEP-YOLOv5s outperforms the existing state-of-the-art methods, offering a robust solution for UAV-based small object detection with enhanced feature extraction and attention-driven adaptability.

## 1. Introduction

With the rapid development of drone technology, it has been gradually applied in various fields, including military and civilian applications. In the field of environmental monitoring, Wang et al. designed ODTDS, a real-time drone-based detection system, for target recognition in complex environments [1]. In the field of dynamics, Sudhanshu Shekhar Jha et al. detected and identified various artificial targets placed in complex imaging geometries with different target backgrounds in different application scenarios, based on drone-based hyperspectral imaging [2]. Rohan et al. proposed a method for detecting and tracking drone targets, including both moving and stationary targets [3]. In the field of smart agriculture, Liao et al. proposed an AI-based agricultural drone to improve the accuracy and efficiency of pesticide spraying and enhance the effectiveness of plant protection [4]. In the field of intelligent inspection, Li et al. designed a drone-based autonomous inspection system based on target detection [5]. In the field of traffic monitoring, Ángel et al. proposed a multi-object tracking (MOT) algorithm for traffic monitoring using drones equipped with optical and thermal cameras [6]. In these applications, drone target detection technology plays a crucial role. Drone target detection refers to the use of high-definition cameras or other sensors mounted on drones to identify, locate, and track specific targets in images or videos using image processing technology and computer vision algorithms. Traditional object detection approaches, which are predominantly based on manually engineered feature extraction techniques [7,8] and handcrafted features [9], demonstrate substantial limitations when applied to aerial imagery. These constraints manifest in several critical aspects: First, the characteristically minute pixel dimensions of objects in aerial images pose significant challenges for conventional handcrafted features to reliably extract discriminative patterns from low-resolution targets, consequently leading to markedly elevated missed detection rates; Second, the distinctive overhead perspective inherent to aerial imaging frequently results in densely clustered objects and severe occlusion scenarios, wherein traditional non-maximum suppression (NMS) algorithms tend to produce erroneous suppression outcomes. Third, the inherent variability in UAV flight altitudes induces pronounced scale variations among targets, fundamentally limiting the effectiveness of fixed-scale sliding window detection paradigms. Furthermore, the complex background artifacts commonly encountered in aerial images—including shadows and regions with homogeneous textures—substantially increase the propensity for false positives in traditional detection frameworks. These compounded challenges underscore the pressing need for novel detection methodologies that can simultaneously address the unique demands of aerial imagery while maintaining computational efficiency, thereby presenting both significant research opportunities and practical applications in the field.

With the rise of deep learning technology, especially the successful application of convolutional neural networks (CNNs) in the field of image recognition, drone target detection technology has ushered in new development opportunities. At present, mainstream target detection algorithms include two-stage detection algorithms based on deep learning (such as the R-CNN series [10], Mask R-CNN [11], and Cascade R-CNN [12]) and one-stage detection algorithms (such as the YOLO series [13,14,15,16], SSD [17] and RetinaNet [18]). These algorithms have achieved great success in the field of natural image detection, but their detection performance significantly decreases when faced with aerial images.

The rest of this paper is organized as follows: Section 2 reviews the relevant literature on drone target detection; Section 3 elaborates on the proposed MEP-YOLOv5s model and introduces the principles of its feature extraction module; Section 4 verifies the superiority of MEP-YOLOv5s through a series of ablation and comparative experiments; and Section 5 summarizes the work of this paper and looks forward to the future research directions.

## 2. Materials and Methods

Many traditional drone target detection methods rely on digital signal analysis methods, such as audio processing and radar signal interpretation. However, these methods exhibit limited accuracy in complex scenarios involving numerous or overlapping targets, and their detection stability degrades significantly in complex environments. Consequently, there has been a growing trend among researchers to adopt deep learning algorithms for extracting object detection features from drone aerial imagery. L Shaodan et al. [19] proposed a segmentation-based detection model (RiceblastSegMask) for rice blast disease detection and resistance assessment, which enables the effective quantification of disease infection levels. H Liang et al. [20] developed a drone-based low-altitude remote sensing system for multi-category concrete bridge damage detection. This system employs a Swin Transformer-based backbone network combined with a multi-scale attention pyramid network and lightweight residual global attention network (LRGA-Net), achieving significant performance improvements in speed and accuracy. B Zhao et al. [21] introduced a cluster analysis-based anchor box optimization strategy to enhance the Faster R-CNN framework’s performance in drone-based marine search and rescue operations. Z Yang et al. [22] presented an effective drone detection framework for infrared imagery. The framework first determines continuous coupling neural network (CCNN) parameters through image statistical features (standard deviation, mean value, etc.), followed by iterative pixel grouping, morphological operations (expansion and erosion), and minimum bounding rectangle extraction to produce final detection results. H Li et al. [23] proposed an improved Mask R-CNN instance segmentation method integrated with hyperspectral sensor technology, enabling the early stage identification of pine wood nematode infections in individual trees. D Wu et al. [24] introduced FSNet, which enhances local and global diversity in forest fire imagery through YOCO data augmentation. The model incorporates a Transformer-based EBBlock module that leverages cross-group interactions to comprehensively extract fire and smoke features while suppressing the background interference. Additionally, FSNet employs a feature pyramid architecture to fuse outputs from four hierarchical stages, significantly mitigating small-target feature loss.

Compared with two-stage drone target detection methods, lightweight single-stage models with a high precision have gained significant traction in specific domains while maintaining competitive detection accuracy. The YOLO series represents a prominent single-stage detection approach [14], with ongoing research focusing on balancing accuracy and efficiency for drone-based applications. J Guo et al. [25] developed a drone aerial detection algorithm based on YOLOv5s, incorporating a more accurate small target detection (MASD) mechanism and multi-scale feature fusion (MCF) path to enhance the feature representation. Y Xu et al. [26] optimized YOLOv5s anchor boxes using K-Means++ clustering and introduced the EIoU loss function to replace the CIoU loss; CIoU enhances DIoU by adding an aspect ratio consistency penalty to jointly optimize the overlap area, center distance, and aspect ratio alignment. EIoU further refines this by decomposing aspect ratio loss into independent width and height components and explicitly modeling center distance with edge length differences. Through finer geometric constraints, EIoU achieves better small-object detection and scale variation handling with improved computational efficiency, while demonstrating an improved detection performance through real-world flight tests. Y Dong et al. [27] designed a lightweight YOLOv4-based drone detection model, enhancing small target detection through improved feature fusion, target augmentation, and candidate box refinement. X Su et al. [28] proposed ASFF Small, an adaptive feature fusion method combining YOLOv3-SPP with ASFF, which is tailored for drone image characteristics. H Tang et al. [29] presented a radar-optical fusion detection method for marine UAVs, effectively addressing the challenges posed by closely spaced overlapping targets in radar imagery.

Contemporary target detection algorithms exhibit distinct technical evolution paths. Two-stage frameworks primarily focus on optimizing feature extraction networks through mechanisms such as multi-scale feature fusion and attention module enhancements. These methods demonstrate a superior performance in complex scenarios (e.g., achieving 46.2% mAP on the COCO dataset [12]) but introduce significant computational overheads due to their hierarchical processing architecture, resulting in an approximately 40% higher inference latency compared with single-stage models (e.g., the delay gap between Faster R-CNN and RetinaNet reaches 41.6% [30]). In contrast, single-stage detectors emphasize module-level innovations, including dynamic sampling strategies, nonlinear activation functions (e.g., Swish [31]), and cross-layer feature interaction mechanisms. However, these improvements often increase the model complexity, with typical single-stage architectures exceeding 45M parameters (e.g., YOLOv5x [15]), complicating deployment on resource-constrained drone platforms.

These technical challenges manifest across three dimensions: (1) Perceptual limitations—drone-acquired images frequently suffer from sub-1080p resolution, high vegetation coverage (>70%), and minute target sizes (<0.05% pixel area); (2) Dynamic environmental interference—flight-induced posture changes and abrupt lighting variations compromise image quality; and (3) Bottlenecks in engineering implementation—algorithms must balance real-time performance and accuracy under hardware constraints. Key limitations include the drone platform’s computing resources (e.g., CPU/GPU computing power, memory bandwidth), power consumption budget, cooling capacity (primarily passive cooling), and payload weight, all of which directly restrict the deployment of complex detection models.

To address these issues, this paper proposes MEP-YOLOv5s—an innovative algorithm for small-target detection in drone aerial imagery.

## 3. Improved Algorithm

### 3.1. Model Structure

This study proposes enhancements to YOLOv5 tailored for the distinct detection challenges that are inherent in drone-captured aerial imagery. To address these challenges, we identify three critical components within the YOLOv5 architecture requiring optimization: feature extraction, loss function formulation, and detection head design. The proposed MEP-YOLOv5s algorithm implements three key modifications:

1. Architectural Redesign: The original C3 module is replaced with a redesigned C2f module integrated with an Exponential Moving Average (EMA [32]) attention mechanism, forming the C2f_EMA module. This configuration optimizes the receptive field geometry to enhance the detection precision for smaller targets.
2. Loss Function Innovation: We replace the traditional CIoU loss function with the improved Modified Probabilistic Distance-IoU (MPDIoU [33]) loss function to enhance the convergence behavior.
3. Detection Head Augmentation: A dedicated small-target detection layer is integrated into the prediction head to strengthen the network’s capability in resolving densely packed minute objects.

**Figure 1:**
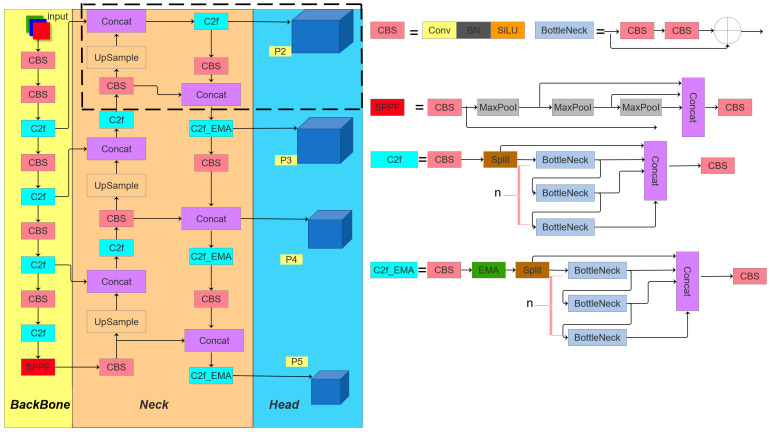
The MEP-YOLOv5s model structure.

The resulting optimized network architecture, termed MEP-YOLOv5s, is visualized in Figure 1.

### 3.2. Feature Converged Network Architecture

#### 3.2.1. Attention-Based Receptive Field Feature Retrieval Module

**Figure 2:**
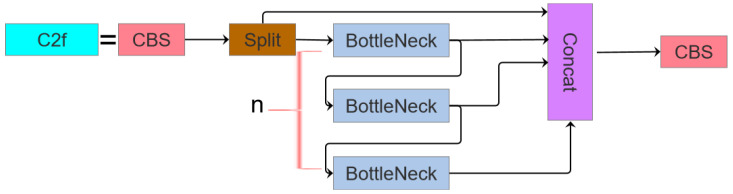
The C2f module structure diagram.

**Figure 3:**
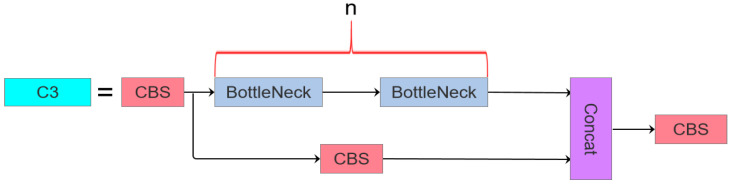
The C3 module structure diagram.

In the YOLOv5 architecture, the C3 module is extensively utilized, whereas the YOLOv8 network incorporates the C2f module. These modules represent distinct structural innovations within their respective frameworks, differing significantly in both functional design and operational principles. The C2f module integrates design philosophies from both the C3 module and ELAN, prioritizing lightweight construction while enhancing the gradient flow information richness. Specifically, it achieves increased model depth and complexity through augmented convolutional layers and connectivity patterns, thereby improving the gradient information capture capabilities. By contrast, the C3 module adopts the split extraction strategy of CSPNet [34], and is integrated with a residual architecture. It comprises two primary components: the CBS main branch gradient module for feature extraction and the Bottleneck module for optimizing computational efficiency by reducing the parametric complexity. The architectural configurations of the C2f and C3 modules are visualized in Figure 2 and Figure 3, respectively.

**Figure 4:**
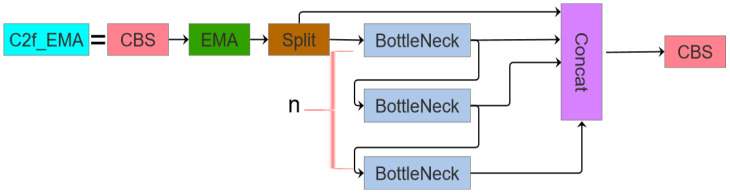
C2f_EMA structure diagram.

As illustrated in the figure, while the C2f module retains the convolutional neural network architecture (functioning as a local feature extractor), the attention mechanism’s strength resides in its global receptive field, enabling the capture of long-range feature dependencies. In this work, we propose C2f_EMA by substituting the C3 module with the C2f module and integrating the EMA attention mechanism within the Neck component (Figure 4).

Assuming that the output tensor of the previous module connected to the C2F_EMA module is ${X\in R}^{C\times H\times W}$, it first passes through the CBS (Convolution-BatchNorm-Silu) module, and then goes through convolution, batch normalization, and the activation function in turn, as shown in Formulas (1)–(3).

(1) $$Conv(X)=W_{conv}*X+b_{conv},$$

(2) $$BN\left(X\right)=\gamma \cdot \frac{Conv\left(X\right)-\mu }{\sqrt{\sigma^{2}+\epsilon }}+\beta ,$$

(3) $$Silu\left(X\right)=X\cdot \sigma \left(BN\left(X\right)\right),$$

Among them, the convolution kernel is $W_{conv}$, the bias is $b_{conv}$, and the number of output channels is $C^{\prime }$. Here, μ and $\sigma^{2}$ are the mean and variance of the batch data, respectively. γ and β are trainable parameters, ϵ is a small constant used to prevent division by zero, and σ(X) is the Sigmoid function. The output from the CBS module is ${X_{CBS}\in R}^{C^{\prime }\times H\times W}$.

Then, after the EMA attention mechanism, $X_{EMA}=EMA(X_{CBS})$. The EMA module outputs the same dimension through multi-scale pooling and the attention weight calculation (see the decomposition below). The Split module splits the EMA output along the channel dimension into two parts:

(4) $$X_{Split1},X_{Split2}=chunk\left(X_{EMA},2\right),$$

The output is $X_{Split1}\in R^{\frac{C^{\prime }}{2}\times H\times W}$, $X_{Split2}\in R^{\frac{C^{\prime }}{2}\times H\times W}$. Next, multiple Bottleneck modules are used to progressively extract the features. Each Bottleneck module consists of multiple convolutional layers and can be configured to use a shortcut connection (residual connection). For the i cycle (i = 1, …, n):

(5) $$X_{Bottleneck}^{\left(i\right)}=\left\{\begin{array}{c} X_{Split2}\;\mathrm{if}\;i=1,\\ Bottleneck(X_{Bottleneck}^{\left(i-1\right)})\;\mathrm{if}\;i>1. \end{array}\right.,$$

(6) $$Bottleneck\left(X\right)=\left\{\begin{array}{c} X+Conv1\left(Conv1\left(X\right)\right)\mathrm{if}\;\mathrm{shortcut}=\mathrm{True}\;\mathrm{and}\;c1=c2,\\ Conv1\left(Conv1\left(X\right)\right)\mathrm{otherwise}.\; \end{array}\right.,$$

Among them, the first Conv1: Conv1 (c, c, k = 3) (the number of channels is unchanged), and the second Conv1: Conv1Conv1 (c, c, k = 3). If residual connections are enabled (shortcut = True), the input X is superimposed. The output $X_{Bottleneck}=R^{\frac{C^{\prime }}{2}\times H\times W}$.

After n Bottleneck processing, we obtain n feature maps $X_{Bottleneck}^{\left(1\right)}$, ....$X_{Bottleneck}^{\left(n\right)}$, as well as the $X_{Split2}$ parts from the initial Split. In the C2f_EMA module, the Concat operation is used to concatenate the feature maps or Bottleneck outputs after the Split along the channel dimension. The concatenation process:

(7) $$X_{Concat}=Concat\left(X_{Split1},X_{Bottleneck}^{\left(1\right)},X_{Bottleneck}^{\left(2\right)},....,X_{Bottleneck}^{\left(n\right)}\right),$$

The output dimension is $X_{Concak}=R^{\frac{C^{\prime }}{2}\times (n+1)\times H\times W}$. The overall process formula is:

(8) $$X_{out}=CBS\left(Concat\left(X_{Split1},X_{Bottleneck}^{\left(1\right)},X_{Bottleneck}^{\left(2\right)},....,X_{Bottleneck}^{\left(n\right)}\right)\right),$$

**Figure 5:**
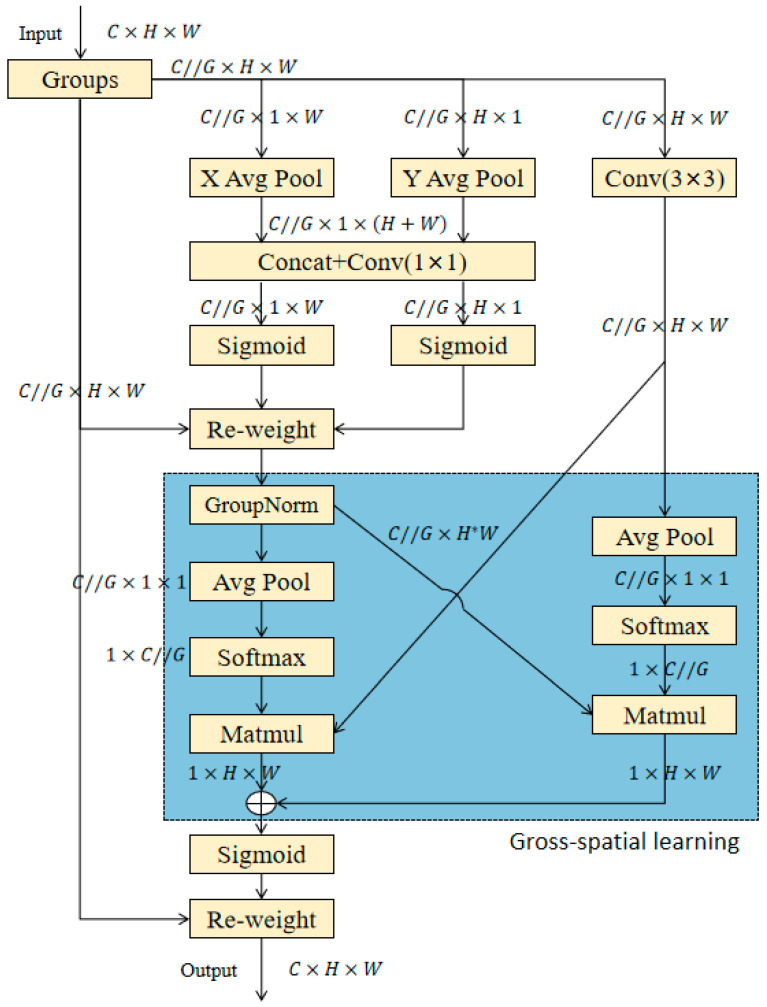
Overall structure of the EMA module.

The EMA [32] module’s architecture is detailed in Figure 5 [32]. To circumnavigate dimensionality reduction issues that are inherent in standard convolutions, the module initially restructures partial channel dimensions into batch dimensions. This is followed by the fusion of output feature maps from two parallel sub-networks, achieved through a local cross-channel interaction mechanism and an innovative cross-spatial learning paradigm. A multi-scale parallel sub-network architecture is implemented to facilitate the collaborative modeling of both short-range and long-range contextual relationships. The implementation workflow proceeds as follows:

1. Feature Encoding and Dimension Preservation:

Inspired by the CA attention mechanism, two encoded features are concatenated along the vertical spatial dimension and processed through a shared 1 × 1 convolution to maintain dimensional consistency. Input tensor: ${X_{CBS}\in R}^{C^{\prime }\times H\times W}$(output from the CBS module), divided into three groups, each with channel number: ${c_{g}=\frac{C}{G}}^{\prime }$. Then the first two groups are horizontally and vertically pooled, respectively:

(9) $$X_{h}=AdaptiveAvgPool2d\left(H,1\right)\left(X_{CBS}\right),X_{W}=AdaptiveAvgPool2d\left(1,W\right)\left(X_{CBS}\right),$$

2. Attention Decomposition and Distribution:

The 1 × 1 convolution output is decomposed into independent vectors, with a 2D binary distribution over linear convolution approximated via a nonlinear Sigmoid activation. The specific process is as follows:

(10) $$hw=Conv1\left(concat\left(X_{h},X_{W}\right)\right),$$

Among them, Conv1 is a 1 × 1 convolution, which is used to fuse the horizontal and vertical information.

(11) $$X_{h}^{\prime },X_{w}^{\prime }=split\left(hw,\left[H,W\right]\right),$$

Then it is weighted by Sigmoid:

(12) $$X_{weighted}=GroupNorm\left(X_{Group}\cdot \sigma \left(X_{h}^{\prime }\right),\sigma \left(X_{w}^{\prime }\right)\right),$$

$X_{Group}$ is the initial grouping tensor, σ(x) is the Sigmoid function, and GroupNorm normalizes each group of features.

3. Dual-Path Attention Aggregation:

(1) 1 × 1 Branch: Intra-group channel attention is aggregated through element-wise multiplication to achieve diversified cross-channel interactions.
(2) 3 × 3 Branch: Local cross-channel interaction information is captured via convolution, augmenting the feature space expressiveness. For the 3 × 3 branch, the 3 × 3 convolution operation is first performed as $X_{Conv3}$, followed by cross-channel interaction attention mechanism fusion, and finally the combined output is performed. The specific process is as follows:

(1) Calculate the global weight of the weighted features $X_{weighted}$ and $X_{Conv3}$ respectively:
  (13) $$Q_{1}=AdaptiveAvgPool2d\left(1,1\right)\left(X_{weighted}\right),$$
  (14) $$Q_{2}=AdaptiveAvgPool2d\left(1,1\right)\left(X_{Conv3}\right),$$
  (15) $$A_{1}=Sigmoid\left(reshape\left(Q_{1},\left(1,c_{g}\right)\right)\right),$$
  (16) $$A_{2}=Sigmoid\left(reshape\left(Q_{2},\left(1,c_{g}\right)\right)\right),$$
(2) Cross attention fusion:
  (17) $$weights=\sigma \left(MatMul(A_{1},Q_{2})+MatMul(A_{2},Q_{1})\right),$$
(3) Adjust the weight shape and act on the grouped features:
  (18) $$X_{OUT}=X_{Group}\cdot reshape\left(weights,\left(1,c_{g}\right)\right),$$

4. Spatial Attention Weighting:

A Sigmoid function generates spatial attention weights, with intra-group output feature maps aggregated to model the pixel-level correlations and emphasize the global contextual significance.

This design paradigm significantly enhances the feature representation capabilities and improves the detection performance across multi-scale targets. By mitigating redundant feature interactions and optimizing multi-scale feature fusion, the EMA mechanism reduces computational complexity while maintaining the advantages of conventional attention mechanisms. This efficient attention framework addresses the limitations of excessive memory consumption and computational overhead associated with traditional approaches.

#### 3.2.2. The Improvement of the Loss Function

The loss function measures the discrepancy between the predicted and ground truth values. A smaller loss value indicates superior model performance. The selection of the loss function is model-specific and is primarily applied during the training phase. The optimization process, which minimizes the loss value to align predictions with the ground truths, hinges on the appropriate selection of the loss function. The selection of the loss function is critical to the model performance. In the YOLOv5s model, the total loss consists of three parts: classification loss, confidence loss, and bounding box loss. Specifically, CIoU loss [35] is employed in bounding box regression, whereas Binary Cross-Entropy (BCE) loss [36] is utilized in confidence and classification tasks. The BCE loss and CIoU loss are calculated as follows:

(19) $$BCE\;loss=-\frac{1}{N}\times \sum_{n=1}^{N}\left[y_{n}\times \log x_{n}+\left(1-y_{n}\right)\times \log\left(1-x_{n}\right)\right],$$

where $y_{n}$ represents the true class, usually taking a value of 0 or 1, $x_{n}$ represents the predicted confidence or target probability obtained using the Sigmoid function, and N is the number of positive and negative samples.

(20) $$CIoU\;loss=1-IoU\left(A,B\right)+\frac{\rho^{2}\left(A_{ctr},B_{ctr}\right)}{C^{2}}+\alpha \nu ,$$

(21) $$\nu =\frac{4}{\pi^{2}}{\left(arctan\frac{\omega^{gt}}{h^{gt}}-arctan\frac{\omega }{h}\right)}^{2},$$

(22) $$\alpha =\frac{\nu }{(1-IoU)+\nu },$$

where IoU represents the Intersection over Union of the areas of the actual and predicted bounding boxes; A and B represent the actual and predicted bounding boxes, respectively; and C represents the length of the diagonal of the smallest rectangle that encloses both A and B. $A_{ctr}$ and $B_{ctr}$ represent the center points of boxes A and B, respectively; α is a positive trade-off parameter; and ν measures the consistency of the aspect ratio. $\rho^{2}(A_{ctr},B_{ctr})$ represents the squared Euclidean distance between $A_{ctr}$ and $B_{ctr}$, and $\omega^{gt}$,$h^{gt}$, ω, and h represent the widths and heights of the actual and predicted bounding boxes, respectively.

**Figure 6:**
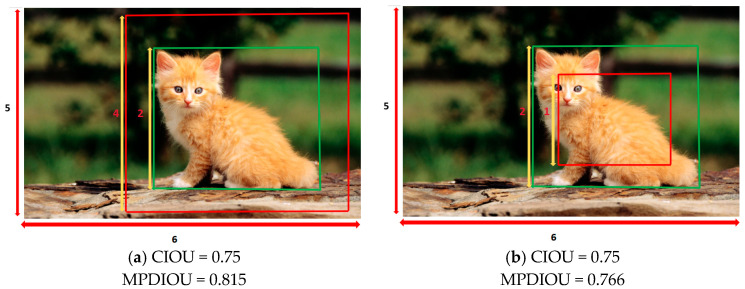
Two cases with different bounding box regression results. Green boxes indicate the true bounding boxes, and red boxes indicate the predicted bounding boxes. The image has a length of 6 and a width of 5. (**a**) Calculate the loss values of CIoU and MPDIoU when a 4 × 4 predicted bounding box fully contains a 2 × 2 ground truth bounding box. (**b**) Calculate the loss values of CIoU and MPDIoU when a 1 × 1 predicted bounding box is fully contained within a 2 × 2 ground truth bounding box.

From Equations (20)–(22), it is evident that when the aspect ratios of the predicted and ground-truth bounding boxes match, the aspect ratio penalty term becomes zero (ν=0). This behavior introduces notable theoretical limitations. By examining the gradients of width (w) and height (h) with respect to the aspect ratio parameter (ν) in the CIoU, we observe that these gradients are opposites. This indicates that w and h cannot be adjusted simultaneously, which is a critical limitation. As shown in Figure 6, the CIoU metric yields identical values for both cases (a) and (b). To mitigate this issue, we introduce MPDIoU [33], a bounding box similarity metric based on the minimum point distance, as an extension of CIoU. This metric optimizes bounding box regression by minimizing the distance between the top-left and bottom-right coordinates of the predicted and ground-truth boxes. The MPDIoU calculation is formally defined in Algorithm 1. As shown in Figure 6, MPDIoU distinguishes between cases (a) and (b).

Algorithm 1 outlines the computational process for MPDIoU:
**Algorithm 1.** Intersection over Union with Minimum Points Distance.**Input:** Two arbitrary convex shapes: $A,B\subseteq S{\subseteq R}^{n}$, width and height of input image: w, h
1: For A and B, $\left(x_{1}^{A},y_{1}^{A}),(x_{2}^{A},y_{2}^{A}\right)$ denote the top-left and bottom-right point coordinates of $A,\;(x_{1}^{B},y_{1}^{B}),(x_{2}^{B},y_{2}^{B})$ denote the top-left and bottom-right point coordinates of B.
2:
$d_{1}^{2}={\left(x_{1}^{B}-x_{1}^{A}\right)}^{2}+{\left(y_{1}^{B}-y_{1}^{A}\right)}^{2}$$,d_{2}^{2}={\left(x_{2}^{B}-x_{2}^{A}\right)}^{2}+{\left(y_{2}^{B}-y_{2}^{A}\right)}^{2}$
3:
$MPDIoU=\frac{A\cap B}{A\cup B}-\frac{d_{1}^{2}}{w^{2}+h^{2}}-\frac{d_{2}^{2}}{w^{2}+h^{2}}$


The Euclidean distance between the top-left vertices of A and B is denoted as $d_{1}^{2}$, and the Euclidean distance between the bottom-right vertices of A and B is denoted as $d_{2}^{2}$. The loss function of MPDIoU can be formulated as:

(23) $$L_{MPDIou}=1-MPDIou=1-\frac{A\cap B}{A\cup B}+\frac{d_{1}^{2}}{w^{2}+h^{2}}+\frac{d_{2}^{2}}{w^{2}+h^{2}},$$

From the preceding formulation, it is evident that MPDIoU quantifies positional and dimensional discrepancies by directly computing the distance between the vertex coordinates of the predicted and ground-truth bounding boxes. As shown in Figure 6, the MPDIoU values differ between cases (a) and (b), while CIOU remains identical. This metric enables effective differentiation between scenarios where the predicted bounding box is inside versus outside the ground truth bounding box, even when maintaining the same aspect ratio. This characteristic ensures accurate bounding box regression and helps to reduce redundant predictions. The loss function depends solely on vertex coordinate distances, with the gradient computation focused exclusively on coordinate offsets, thereby eliminating mutual dependencies from the aspect ratio parameters. This enables the independent adjustment of width and height via coordinate optimization, mitigating gradient conflicts. As shown in Equation (24), all the factors considered in existing loss functions—such as the area of the non-overlapping regions, distance between the center points, width discrepancy, and height discrepancy—can be determined through the coordinates of the top-left and bottom-right corners. This implies that MPDIoU not only incorporates these evaluation dimensions but also streamlines the computational process by using corner coordinates as the sole input for regression.

(24) $$\left\{\begin{array}{c} \left|C\right|=(max(x_{2}^{gt},x_{2}^{prd})-min(x_{1}^{gt},x_{1}^{prd}))*(max(y_{2}^{gt},y_{2}^{prd})-min(y_{1}^{gt},y_{1}^{prd}))\\ x_{C}^{gt}=\frac{x_{1}^{gt}+x_{2}^{gt}}{2},y_{C}^{gt}=\frac{y_{1}^{gt}+y_{2}^{gt}}{2},x_{C}^{prd}=\frac{x_{1}^{prd}+x_{2}^{prd}}{2},y_{C}^{prd}=\frac{y_{1}^{prd}+y_{2}^{prd}}{2}\\ w_{gt}=x_{2}^{gt}-x_{1}^{gt},h_{gt}=h_{2}^{gt}-h_{1}^{gt},w_{prd}=x_{2}^{prd}-x_{1}^{prd},h_{prd}=h_{2}^{prd}-h_{1}^{prd} \end{array}\right.,$$

where the four points ($x_{1}^{gt},y_{1}^{gt}$), ($x_{2}^{gt}$,$x_{2}^{gt}$), ($x_{1}^{prd},y_{1}^{prd}$), and ($x_{2}^{prd},y_{2}^{prd}$) represent the coordinates of the top-left and bottom-right points between the actual and predicted bounding boxes, respectively. $\left|C\right|$ represents the area of the smallest rectangle that encloses both boxes A and B. The points ($x_{C}^{gt},y_{C}^{gt}$) and ($x_{C}^{prd},y_{C}^{prd}$) represent the coordinates of the center points of the actual and predicted bounding boxes, respectively. $w_{gt}$, $h_{gt}$, $w_{prd},$ and $h_{prd}$ represent the widths and heights of the actual and predicted bounding boxes, respectively.

#### 3.2.3. Small Target Detection Layer

As illustrated in Figure 1, the newly introduced P2 detection branch (highlighted by the dashed box) is tailored for the detection of extremely small objects. Through a cross-level feature fusion mechanism, this branch efficiently integrates low-level features derived from shallow convolutional layers. These features preserve critical spatial structural details, including edge contours and texture patterns.

(25) $$H_{I}=\left[\frac{H_{input}}{2^{I}}\right](I=number\;of\;layers)$$

However, as demonstrated by the feature map resolution formula in Equation (8), successive convolutional and pooling operations progressively reduce the feature map resolution with increasing network depth. This resolution loss has dual consequences: (1) deep features may sacrifice the geometric details of small objects (e.g., diminutive pedestrians and vehicles); and (2) channel attention mechanisms may overemphasize large-object semantics, exacerbating false/missed detections. By fusing the shallow features, the P2 branch preserves object geometric integrity, enhances bounding box regression precision for objects < 16 × 16 pixels, and consequently improves the overall detection performance. Each branch is shown in Table 1.

## 4. Experiment and Result Analysis

### 4.1. Experimental Setup

The hardware environment configuration used in the experiment is as follows: the central processing unit (CPU) is Intel(R)Core (TM)i5-12500T, and the graphics processing unit (GPU) is NVIDIA GeForceGTX3090 (equipped with 24 GB of video memory). The software environment includes CUDA11.2 and Python3.8. During the experiment, unified training parameters were used, and the model performance was further optimized through fine-tuning. The specific parameter settings are detailed in Table 2.

#### 4.1.1. Experimental Datasets

**Figure 7:**
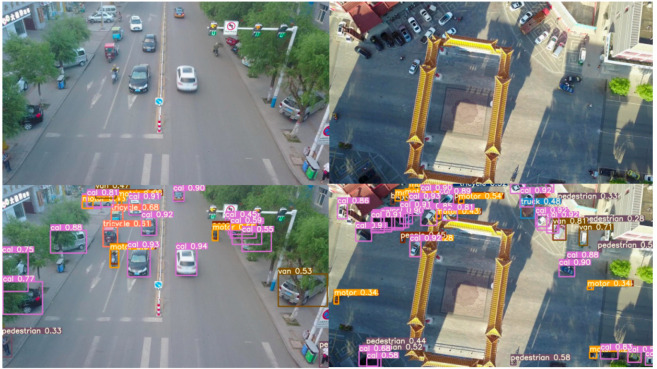
Randomly selected images from the VisDrone-2019 dataset.

**Figure 8:**
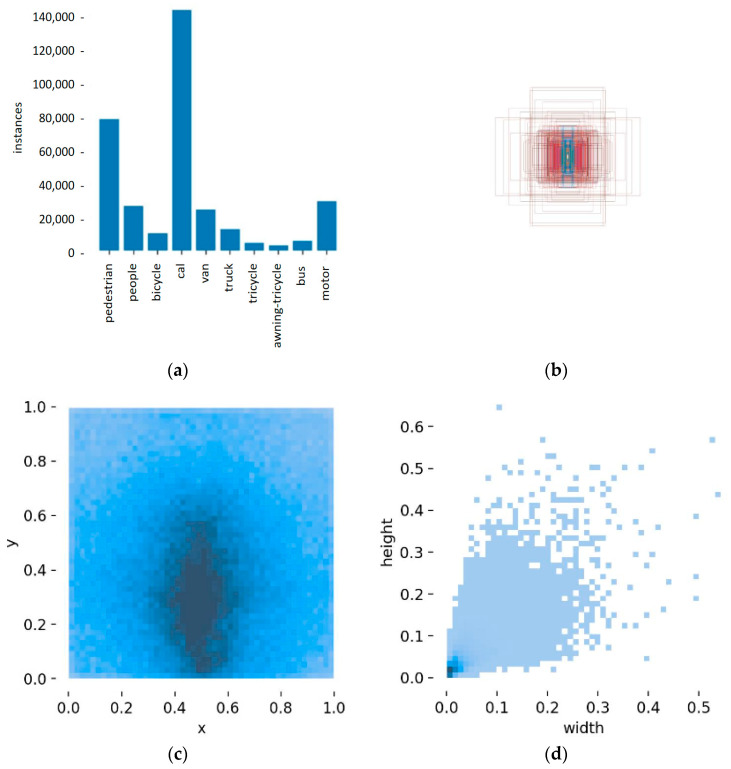
The class and bounding box information for each detected object. (**a**) Number of instances per category. (**b**) Bounding box dimensions and quantities. (**c**) Center point coordinates of the bounding boxes. (**d**) Length and width of the bounding boxes.

The experiments employed the VisDrone2019 benchmark—a large-scale drone-captured dataset specifically designed for small-object detection—publicly released by research teams, including Tianjin University. This dataset comprises 6471 training samples, 548 validation samples, and 1610 testing samples, all annotated across 10 distinct object categories. Figure 7 displays representative samples from the dataset, featuring ground-truth annotations of four fundamental transportation categories: pedestrians, cars, motorcycles, and vans. The visual examples demonstrate typical object distribution patterns and scale variations in urban surveillance scenarios. Figure 8 represents the class and bounding box information for each detected object, where Figure 8a shows the data volume of the training set and the number of each category; Figure 8b illustrates the size and quantity of the bounding boxes; Figure 8c depicts the position of the center point relative to the entire image; and Figure 8d presents the aspect ratio of the target relative to the entire image.

#### 4.1.2. Experimental Evaluation Metrics

This study adopts Precision (P), Recall (R), F1 score, mean average precision (mAP@0.5), and mAP@0.5:0.95 as the evaluation metrics. Among these, mAP@0.5 is designated as the primary evaluation criterion. The definitions are elaborated as follows:

1. Precision (P) is defined as the proportion of true positive samples among those predicted as positive by the model. The calculation formula is:
  (26) $$P=\frac{TP}{TP+FP},$$
  where TP represents True Positive, i.e., the number of samples correctly predicted as positive by the model, and FP represents False Positive, i.e., the number of samples incorrectly predicted as positive by the model.
2. Recall (R) represents the proportion of true positive samples that are correctly predicted, and the calculation formula is:
  (27) $$R=\frac{TP}{TP+FN},$$
  where FN represents False Negative, i.e., the number of samples incorrectly predicted as negative by the model.
3. F1 score (also known as the F-score or F-measure) is the harmonic mean of Precision and Recall, serving as a balanced evaluation metric that combines both a model’s precision and recall capabilities, and the calculation formula is:
  (28) $$F1=\frac{2}{\frac{1}{P}+\frac{1}{R}}=2\times \frac{P\times R}{P+R},$$
  where P and R, respectively, denote Precision and Recall.
4. The Average Precision (AP) for a single class is calculated by interpolating and integrating the precision–recall curve, and the formula is:
  (29) $$AP=\int_{0}^{1}P(R)dR,$$
  where P (R) represents the precision at recall rate R.
5. The mean Average Precision (mAP) is the average of AP across all the classes, and the calculation formula is:
  (30) $$mAP=\frac{1}{N}\sum_{i=1}^{N}{AP}_{i},$$
  where N represents the total number of classes, and ${AP}_{i}$ represents the average precision of the i-th class.

Specifically, mAP@0.5 denotes the mean average precision calculated with an Intersection-over-Union (IoU) threshold of 0.5, while mAP@0.5:0.95 represents the average mAP across ten discrete IoU thresholds (0.5 to 0.95, in 0.05 increments) to systematically assess the model performance under varying localization precision requirements.

To address the computational constraints of deploying drone-based systems on mobile devices, we introduce a Comprehensive Performance Indicator (CPI).

(31) $$CPI=\left(\alpha \times mAP@0.5+\left(1-\alpha \right)\times \frac{1}{GFLOPS}\right)\times 100,$$

As can be seen from Equation (31), the CPI takes into account both detection accuracy and inference speed. Here, α is the weighting coefficient, $\alpha \in \left[0,1\right]$, with a default value of 0.5. When α > 0.5, greater emphasis is placed on the detection accuracy; when α < 0.5, greater emphasis is placed on the inference speed.

### 4.2. Contrast Experiment

#### 4.2.1. Results of the MEP-YOLOv5s Experiments

**Figure 9:**
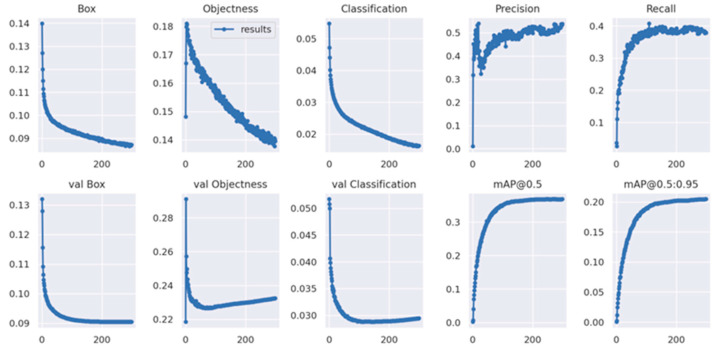
YOLOv5s plot of the training results.

**Figure 10:**
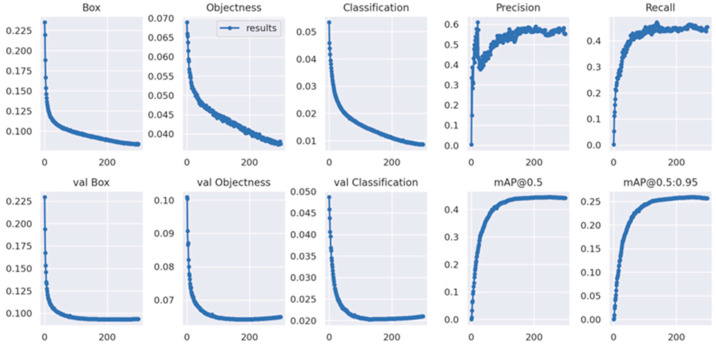
The MEP-YOLOv5s training results plot.

To quantitatively validate the performance enhancement of the proposed model, a comparative analysis was conducted between MEP-YOLOv5s and the baseline YOLOv5s architecture. Figure 9 and Figure 10 present the training results of the two models on the VisDrone-2019 dataset. During the model training, the dynamic evolution of loss values is a key indicator of learning efficiency. The experimental results show that MEP-YOLOv5s demonstrates significant advantages over the baseline YOLOv5s model. Specifically, MEP-YOLOv5s exhibits a steeper initial decline in localization loss (Box Loss), indicating a faster convergence to a high-precision solution space in the bounding box regression tasks. This confirms the improved architecture’s enhanced spatial feature parsing capability, particularly in geometric constraint modeling. The quantitative analysis of confidence loss (Objectness Loss) shows that MEP-YOLOv5s reduces background–foreground classification uncertainty through multi-scale feature fusion, maintaining consistently lower loss values than the baseline and demonstrating more robust target presence judgment. However, when observing the baseline model, it is found that it first shows a straight upward trend and then declines. This indicates that the objectness loss is overfitting. At this point, the obj or obj_pw hyperparameters should be reduced to decrease the contribution of the objectness loss to the total loss. The classification loss (Classification Loss) trajectory further supports this, with MEP-YOLOv5s achieving early and sustained loss reduction, attributed to its attention enhancement module that boosts the category discrimination confidence via cross-channel feature calibration.

Precision (Precision) dynamics, measuring positive detection accuracy, reveal the improved generalization capability of MEP-YOLOv5s. While the baseline YOLOv5s shows stable but fluctuating precision, MEP-YOLOv5s achieves significant late-stage precision gains, especially in complex scenarios, indicating superior false positive suppression. This late-stage surge is linked to its progressive feature refinement mechanism, which dynamically adjusts feature pyramid contributions to enhance the final detection box quality.

Recall (Recall), assessing instance coverage, highlights differences in object retrieval between models. Despite fluctuations, MEP-YOLOv5s periodically surpasses the baseline, indicating enhanced recall in challenging samples while maintaining precision, which is valuable in dense or small-object detection.

In the detection quality assessment, MEP-YOLOv5s shows clear advantages in mAP@0.5 (IoU threshold 0.5), with a 4.2% higher mAP than the baseline, indicating better detection box screening under relaxed standards via optimized NMS. Under a stricter mAP@0.5:0.95 evaluation, MEP-YOLOv5s still achieves higher scores, validating the multi-scale feature enhancement module’s robustness in complex scenarios.

A systematic comparison confirms the comprehensive improvements in localization accuracy, classification confidence, and false positive control of MEP-YOLOv5s through architectural innovations. Its training loss curves, precision–recall balance, and multi-scale detection performance collectively demonstrate its strong adaptability to diverse detection scenarios. For practical deployment, MEP-YOLOv5s enhances the detection reliability in complex environments while preserving the real-time performance of YOLO, making it suitable for accuracy-critical applications like autonomous driving and intelligent surveillance.

Quantitative evaluations reveal that MEP-YOLOv5s outperforms the baseline model across all metrics: (1) achieving a 2.5% absolute gain in small-object detection P (Precision), (2) demonstrating a 19.5% relative improvement in R (Recall), (3) increasing mAP@0.5 by 20.1%, and (4) showing a 19.07% absolute enhancement in the CPI (Comprehensive Performance Index). The detailed results are documented in Table 3.

#### 4.2.2. Effect Diagram

**Figure 11:**
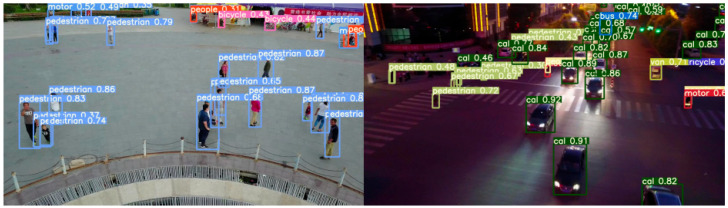
Test result diagram of YOLOv5s.

**Figure 12:**
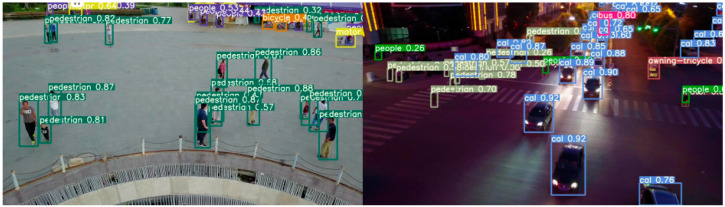
Test result diagram of MEP-YOLOv5s.

Figure 11 and Figure 12 present the detection results of the YOLOv5s model and the MEP-YOLOv5s model under both daytime and nighttime conditions. The schematic diagrams in these figures demonstrate a higher level of detail, reflecting the algorithmic advancements achieved. A comparison of the two figures reveals that the MEP-YOLOv5s model exhibits certain advantages in detecting small targets. This enhancement effectively mitigates the challenges that are typically associated with missed detections and false alarms, thereby improving the overall detection accuracy of the network.

Upon closer observation, it can be noted that during the nighttime, some small-pixel targets exhibit confidence scores below 0.4. Even our improved model struggles to avoid this issue, which is attributed to the degradation in image resolution caused by the reduced ambient lighting. To address this problem in future work, integrating infrared images for detection presents a promising direction.

#### 4.2.3. Ablation Experiment

Ablation studies were conducted to rigorously validate the efficacy of individual algorithmic enhancements. Based on the YOLOv5s baseline, systematic evaluations were performed to quantify the performance contributions of each modified component within the MEP-YOLOv5s architecture. As detailed in Table 4, modular components were incrementally integrated while maintaining consistent optimization strategies and hyperparameter configurations. This controlled approach enabled the precise observation of metric variations across: (1) Precision (P), (2) Recall (R), (3) F1 (F1 score), (3) mAP@0.5, (4) mAP@0.5:0.95, and (5) the Comprehensive Performance Index (CPI).

Three architectural modifications were implemented: (1) Incorporation of a 3× downsampling small-object detection head into the original YOLOv5s architecture; (2) substitution of the Backbone’s C3 module with C2f, and replacement of the Neck’s C3 module with C2f-EMA; and (3) adoption of the MPDIoU loss function. The empirical findings demonstrate that: (1) The added P2 detection head yielded an 18.2-percentage-point absolute improvement in mAP@0.5, confirming that size-adaptive anchor boxes significantly mitigate missed detections due to scale discrepancies; (2) The C2f-EMA module produced an additional 18.75-percentage-point enhancement in mAP@0.5, substantiating the attention mechanism’s capability to augment feature discriminability; (3) The implementation of MPDIoU resulted in a 4% precision reduction compared with the previous configuration. This is primarily attributed to differences in the gradient characteristics between MPDIoU and CIOU, where the original learning rate led to training instability. Additionally, the weighting between MPDIoU and the other loss components (classification loss and confidence loss) requires rebalancing. However, under the same computational budget, it achieved relative improvements of 3.3% in Recall and 1.83% in mAP@0.5, demonstrating the effectiveness of cross-layer feature fusion in enhancing small-object detection capability.

### 4.3. Comparison with the Other Models

To evaluate the methodological contributions of MEP-YOLOv5s, we conducted comparative analyses against seven state-of-the-art object detection frameworks—Faster-RCNN, YOLOv3, YOLOv5s, YOLOv5m, YOLOv5L, YOLOv7, and YOLOv8—under identical experimental protocols. As presented in Table 5, comprehensive evaluations on the VisDrone-2019 benchmark demonstrate the superior performance of MEP-YOLOv5s across both precision (P) and recall (R) metrics. Notably, the model achieved absolute mAP@0.5 gains of 12.94, 17.72, 20.9, 24.30, 17.72, 11.25, and 8.53 percentage points over the comparative baselines, respectively, with the most substantial enhancement (20.9 percentage points) observed against YOLOv5s. Furthermore, MEP-YOLOv5s achieved the highest Comprehensive Performance Indicator (CPI, α = 0.5) score of 22.69, surpassing all the comparative models in this metric.

### 4.4. Comparative Experiments on the Other Datasets

To validate the robustness and practical applicability of the proposed methodology, the MEP-YOLOv5s model underwent testing on the NWPU VHR-10 dataset. This dataset, originally published by Northwestern Polytechnical University, comprises 800 high-resolution remote-sensing images spanning ten distinct categories of geospatial objects. To assess the model’s generalization capability, a comparative analysis was conducted between MEP-YOLOv5s and alternative one-stage detection frameworks utilizing the identical NWPU VHR-10 dataset. The experimental configuration maintained consistent training parameters and evaluation metrics as previously outlined, with the detailed findings presented in Table 6.

With an mAP@0.5 of 90%, the MEP-YOLOv5s model demonstrated significant performance superiority over the established benchmark models, including the YOLOv5s baseline architecture and larger-scale variants, such as YOLOv5m and YOLOv5l. Specifically, the model achieved mAP@0.5 values that were 0.020, 0.0089, and 0.0192 higher than those of the comparable YOLOv5m, YOLOv5l, and YOLOv5s frameworks, respectively. This quantitative enhancement underscores the model’s capacity to maintain superior detection accuracy and generalization competence across diverse datasets.

Furthermore, within the UAV-captured scene dataset, the Comprehensive Performance Indicator (CPI) of the MEP-YOLOv5s framework exceeded that of the equivalently scaled models, thereby providing further empirical validation of the proposed enhancement strategies. Systematic ablation studies and comparative experiments confirmed that each innovative module introduced in this work—specifically the C2f_EMA mechanism and MPDIoU loss function—contributed positively to optimizing the YOLOv5 architecture. These findings collectively substantiate the operational viability of deploying the MEP-YOLOv5s model on UAV platforms for the efficient execution of multi-category object detection tasks.

## 5. Summary and Expectation

This paper proposes an efficient and practical object detection model based on YOLOv5, specifically addressing the challenges that are inherent in small object detection within UAV aerial imagery. To enhance the detection accuracy while maintaining real-time UAV detection capabilities, three effective improvement strategies are presented. First, to mitigate the difficulties in identifying small-scale objects in UAV-captured images, an additional detection layer was incorporated into the feature fusion network, thereby augmenting the model’s sensitivity to fine-grained features. Second, we developed a novel feature extraction module, termed C2f_EMA, which replaces the conventional C3 module with a modified C2f architecture and integrates the Exponential Moving Average (EMA) attention mechanism in the Neck component. This innovation demonstrably enhances the model’s capability to detect distant and diminutive objects. Third, we introduce the MPDIoU loss function to simultaneously optimize localization precision and classification accuracy. Concurrently, a novel evaluation metric, termed CPI, was designed to harmonize detection accuracy with inference speed. The experimental results across two benchmark datasets—VisDrone2019 and NWPU VHR-10—demonstrate that our proposed MEP-YOLOv5s model achieves a superior performance compared with the baseline YOLOv5, with significant improvements observed in the precision (P), mAP@0.5, and CPI metrics. These advancements underscore the model’s deployment potential in UAV-based object detection applications.

Future research directions will focus on advancing UAV aerial object detection toward lightweight and intelligent paradigms. By integrating advanced technologies, such as multi-source data fusion, self-supervised learning frameworks, and edge computing architectures, it is anticipated that real-time detection systems with enhanced accuracy and reduced latency can be realized in complex operational scenarios. Such developments will provide robust technical support for applications in smart cities, environmental monitoring, and disaster-response missions.

## Figures and Tables

**Table 1:** The anchor point setting for each detection branch.

Detection Branch	Anchor Frame Configuration
P2	(4,5), (8,10), (22,18)
P3	(10,13), (16,30), (33,23)
P4	(30,61), (62,45), (59,119)
P5	(116,90), (156,198), (373,326)

**Table 2:** Experimental parameters.

Parameter Name	Parameter Setting
batch size	16
learning rate	0.01
size of the image	640 × 640
number of iterations	300
network depth, network width	0.8, 1

**Table 3:** Comparison of training results.

Model	Precision (%)	Recall (%)	F1 (%)	mAP@0.5 (%)	mAP@0.5:0.95 (%)	GFLOPS	CPI		
							α = 0.25	α = 0.5	α = 0.75
YOLOv5s	53.9	37.8	44.43	36.8	20.5	93.1	10.00	18.93	27.86
MEP-YOLOv5s	55.3	45.2	49.74	44.2	25.7	112.4	11.71	22.54	33.37

**Table 4:** Ablation test results.

Model	Precision (%)	Recall (%)	F1 (%)	mAP@0.5 (%)	mAP@0.5:0.95 (%)	GFLOPS	CPI		
							α = 0.25	α = 0.5	α = 0.75
YOLOv5s	53.2	38.1	44.40	36.8	20.5	93.1	10.00	18.93	27.86
+P2	57	43.9	49.59	43.5	24.8	104.1	11.59	22.23	32.86
+C2f_EMA	57.3	44.3	49.96	43.7	25.3	112.4	11.59	22.29	32.99
+MPDIOU	55	45.9	50.03	44.5	25.9	112.4	11.79	22.69	33.59

**Table 5:** Comparison of MEP-YOLOv5s and other models on the VisDrone-2019 dataset.

Model	Precision (%)	Recall (%)	F1 (%)	mAP@0.5 (%)	mAP@0.5:0.95 (%)	GFLOPS	CPI		
							α = 0.25	α = 0.5	α = 0.75
Faster-RCNN	-	-	-	39.4	23	251.4	10.14	19.89	29.64
YOLOv3	51.8	39	44.49	37.8	20.8	154.7	9.93	19.22	28.51
YOLO5s	53.2	38.1	44.40	36.8	20.5	93.1	10.00	18.93	27.86
YOLOv5m	51.9	37.1	43.26	35.8	19.9	50.7	10.42	18.88	27.34
YOLOv5L	54.1	38.8	45.19	37.8	21.5	114.1	10.10	29.33	28.56
YOLOv7	51.6	41.3	45.87	40	22.8	103.3	10.72	20.48	30.24
YOLOv8	51.5	40.1	45.09	41	24.4	165.7	10.70	20.80	30.90
MEP-YOLOv5s	55	45.9	50.03	44.5	25.9	112.4	11.79	22.69	33.59

**Table 6:** Comparing the experimental results of MEP-YOLOv5s and other models on the NWPU VHR-10 dataset.

Model	Precision (%)	Recall (%)	F1 (%)	mAP@0.5 (%)	mAP@0.5:0.95 (%)	GFLOPS	CPI		
							α = 0.25	α = 0.5	α
Faster-RCNN	-	-	-	74.6	47.6	251.4	18.94	37.49	56.04
YOLOv3	94.9	83.5	88.83	89.7	60.9	154.7	22.90	45.17	67.43
YOLOv5s	95.9	83.5	89.27	88.3	59.6	93.1	22.88	44.68	66.49
YOLOv5m	93.6	83.4	88.20	88.2	59.2	50.7	23.52	45.08	66.64
YOLOv5L	93.6	84.4	88.76	89.2	60.4	114.7	22.95	45.03	67.11
YOLOv7	94.6	82.3	88.02	89.6	56	103.3	23.12	45.28	67.44
YOLOv8	90.4	82	85.99	86.7	57.9	165.7	22.12	43.65	65.17
MEP-YOLOv5s	93.0	84.2	88.38	90.0	59.1	112.4	23.16	45.44	67.72

## Data Availability

The publicly accessible VisDrone-2019 and the NWPU VHR-10 datasets are available for download from the following links: https://github.com/VisDrone/VisDrone-Dataset (accessed on 20 March 2025) and https://github.com/Gaoshuaikun/NWPU-VHR-10 (accessed on 10 November 2024), respectively. Publicly available web data were used in this study and can be accessed without restrictions.
